# To Advance AI Use in Education, Focus on Understanding Educators

**DOI:** 10.1007/s40593-023-00351-4

**Published:** 2023-06-09

**Authors:** René F. Kizilcec

**Affiliations:** Department of Information Science, Cornell Univeristy, Ithaca, NY USA

**Keywords:** Educators, Psychology, Technology acceptance, Trust, Implementation, Predictive analytics

## Abstract

A better understanding of educators’ perspectives of emerging education technology, specifically tools that incorporate AI, is essential to unlock the full potential benefits of these innovations. While prior research has primarily emphasized technological advancements, it has overlooked the profound influence of social, psychological, and cultural factors in shaping educators’ perceptions, trust, and adoption of educational technology. As increasingly powerful AI tools emerge, their design must be rooted in a deep understanding of educators' needs and perspectives. It is only with the acceptance and trust of educators that these innovative solutions can elevate learning outcomes, academic achievements, and educational equity.

How educators view and intend to use emerging education technology in schools and homes is fundamentally shaped by social-psychological and contextual factors. Most research on Artificial Intelligence in education (AIED) has focused on technological improvements (e.g., creating adaptive or personalized systems, creating more accurate and fair algorithms) (Zawacki-Richter et al., 2019). In contrast, studies of why real-world implementations of education technologies have failed emphasize the role of social, psychological, and cultural issues (Ames, 2019; Cuban, 2009; Reich, 2020). This calls for more research of education technology from a psychological perspective to understand what factors shape the way educators perceive, trust, and use education technology in teaching practice. These new theoretical insights hold promise for advancing the design and implementation of AIED interventions in the field to encourage their adoption and effective use to ultimately improve learning outcomes, academic attainment, and educational equity (Buckingham Shum, Ferguson, & Martinez-Maldonado, 2019).

Technology has become an integral part of learning and teaching practice for millions of students and working adults around the world. In the 2018 PISA survey, 71% of US students reported using laptops in classrooms (Bryant et al., 2020). Middle and high school students use tutoring systems such as Carnegie Learning for mathematics, science, and English classes, homework systems such as ASSISTments for practicing mathematics problems, and many other education technologies to create, collaborate, practice, and share their work with others. College students and most working learners use learning management systems such as Canvas that offer increasingly sophisticated content features. The COVID-19 pandemic rapidly accelerated this trend by exposing students and teachers at every level to new online learning tools and practices, which has encouraged the adoption and implementation of education technologies over the next decade (Reich & Mehta, 2020). In early 2023, the sudden popularity and wide accessibility of generative AI tools based on large language models, such as OpenAI’s ChatGPT, has forced educators as well as academic policy makers to once again pay attention to how technology is used in education (Yan et al., 2023).

The pervasive use of technology in education, and the detailed information it records on student activity and performance, means that educators can get insights into students’ progress and struggles. This feedback to educators is increasingly being provided by Predictive Learning Analytics (PLA) that leverage machine learning and Artificial Intelligence methods to surface predictions about which students are at risk of underperformance or dropout. For example, in 2022, 20% of all K-12 schools in the United States, which is 25k schools that serve 10 m students, use BrightBytes, one of many big data platforms with PLA capabilities that show educators risk predictions for their students (Baker et al., 2020). Likewise, many higher education institutions have adopted PLA to identify students at risk of dropping out of college. For example, the Signals system at Purdue University began back in 2006 (Arnold & Pistilli, 2012), the GPS Advising system at Georgia State University started in 2012 (Kurzweil & Wu, 2015), and OU Analyse at the Open University started in 2014 (Kuzilek et al., 2015). Since then, various commercial systems have entered the market to provide PLA-based insights to administrators and educators.

A large share of the research on education technology has focused on the technological implementation of these increasingly data-driven systems. Research communities including AIED, educational data mining, and learning analytics have been advancing the state of the art in PLA, for instance by pushing the boundaries on prediction models of student learning or affect. However, this work fails to address the issues identified by seminal studies on why classroom technology has not had the transformative impact on education and learning outcomes that some prominent scholars predicted (Christensen, Horn & Johnson, 2008). These studies do not point to any technical shortcomings as the culprit: the reason why classroom computers were *Oversold & Underused*, according to Cuban (2009), *One Laptop Per Child* … *died*, according to Ames (2019), and recent technological innovations such as MOOCs were a *Failure to Disrupt*, according to Justin Reich (2020), is not related to their technical features (e.g., their prediction accuracy, or data analytics and visualization capabilities). Instead, they all highlight the role of social, psychological, or cultural issues in how stakeholders relate to a given education technology, especially their perceptions, trust, and intentions towards using it. In the case of OU Analyse, a recent evaluation study showed that if educators actively use the PLA, it can lead to substantial gains for disadvantaged students; but only a fraction of educators are willing to use the system and many of them are unable to use it effectively (Herodotou et al., 2019; Hlosta et al., 2021).

These insights ought to motivate more research into the critical role of social-psychological, cultural, and contextual factors that influence educators’ adoption of technology, especially AI-powered technologies like PLA that are prone to misconceptions and fears about employment and privacy risks (Nazaretsky et al., 2022a, b). Educators’ trust in and effective use of PLA are instrumental for realizing their potential benefits, and advances in explainable AI in education are beginning to offer educators new opportunities to understand the recommendations that PLA systems provide (Khosravi et al., 2022). Efforts to make AI models explainable to people matter. In fact, a recent synthesis of six major initiatives to establish ethical principles for the adoption of socially beneficial AI has identified a core set of five ethical principles for AI systems: it includes the four core bioethics principles of beneficence, non-maleficence, autonomy, and justice, plus the addition of explicability, which is a combination of intelligibility (i.e., how the PLA system works) and accountability (i.e., who is responsible for how it works) (Floridi & Cowls, 2022). Explainable AI in education is therefore more than just a useful new feature, it is an ethical design choice. And yet, it is unclear if educators’ resistance to adopting PLA can be traced back to a lack of explainable AI, or if it is related to a more fundamental process, such as algorithm aversion.

Algorithm aversion is the phenomenon that characterizes people’s negative attitudes towards using algorithms and it influences how they respond to AI systems in real-world environments (Dietvorst, Simmons, & Massey, 2015). People tend to trust humans more than algorithms and follow human recommendations more, especially if the task is considered subjective or it requires attention to individual uniqueness. At-risk predictions performed by PLA systems arguably require close attention to individual learner characteristics, which raises the likelihood of algorithm aversion from educators. This could lead to irrational responses, such as punishing the AI system more than a human for making the same mistake. An educator might therefore be less inclined to give a PLA system the benefit of the doubt when it errs; they would judge the system to be less competent and quickly lose trust. The following theoretical models can offer a better understanding of the influence of educators’ perceptions of a PLA system on their attitudes and actions.

## Technology Acceptance, Academic Resistance, and Trust

The Technology Acceptance Model (TAM) introduced by Davis (1989) helps to disentangle and understand how an individual’s perceptions of a technology influence their attitudes and behavioral intentions, which in turn affect their actual use of the technology (Yi & Hwang, 2003). The model considers two kinds of perceptions as fundamental determinants of user acceptance of technology: the perceived usefulness of a technology and its perceived ease of use. TAM has been a widely used and well-performing predictive model of technology adoption in a large variety of organizational and personal contexts (Adams, Nelson & Todd, 1992; Venkatesh and Davis, 2000; Lee et al., 2003; Venkatesh and Bala, 2008). According to TAM, the perceptions that users have about a technology are not only critical determinants of technology acceptance and actual use, but those perceptions are also malleable. A technology’s presentation, framing, design, marketing, among other factors, can influence perceptions of usefulness and ease of use. As one of the first models to incorporate psychological factors in technology acceptance, TAM provides a strong foundation for developing the psychology of technology in education because it clearly lays out a mechanism by which malleable perceptions affect attitudes and behavioral intentions.

While several studies have applied TAM in the context of education (e.g., Fathema et al., 2015; Park, 2009; Teo, 2009), these studies almost exclusively use structural equation modeling to apply TAM to collected survey data. Prior work on technology acceptance in education therefore does not tend to experimentally investigate changes that have a causal effect on education technology acceptance, even though TAM is a valid and predictive model of technology adoption in education (Teo, 2009). This provides a strong foundation for using TAM to develop a causal understanding of how perceptions and behavioral intentions about education technology are affected by framing, presentation, transparency, training, and context. However, TAM does not account for the strong emotional responses that new technologies, especially ones powered by AI, can trigger among educators. The Academic Resistance Model (ARM) introduced by Piderit (2000) accounts for cognitive, emotional, and intentional attitudes about an organizational change, which complements TAM. For example, Rienties (2014) found that even though most educators cognitively appreciated the usefulness of a new student evaluation system, they felt strong negative emotional attitudes and resisted the change due to anxiety and mistrust.

Educators’ trust in a technology plays a critical role in the adoption of education technology (Cukurova et al., 2020). Their level of trust depends on accessible evidence that the technology is trustworthy, such as an endorsement from close colleagues, expert researchers, or a reputable organization. It also depends on educators’ cognitive and emotional responses to the technology: for example, different framings of a technology can affect how much teachers trust it (Nazaretsky, Ariely et al., 2022), and different levels of algorithm transparency can affect how much students trust it (Kizilcec, 2016). This highlights the impact of how an AI system is framed to educators and how much transparency (or other types of explainability) is provided.

## Beneficiary Framing, Algorithm Transparency and Literacy

If people’s perceptions of a technology are critical determinants of their eventual use, then it is important to understand how their perceptions can be strategically influenced. Research on persuasion has shown that a message resonates more with an audience if it is relevant to the audience’s perspective (Cialdini, 2003; Clary & Snyder, 1999; Rothman & Salovey, 1997). Educators have a unique perspective on the use of education technology and carefully consider the consequences of its use: they care about how the technology can complement their efforts but does not replace them (educator benefits), or how much it can help improve students’ outcomes (student benefits). For example, in a clinical context, Grant and Hoffmann (2011) found that doctors practiced better hand hygiene if signs emphasized patient safety instead of doctor safety. While it may seem like a small difference, several studies have shown that small changes in the content of messages can produce large changes in people’s mindsets and behaviors (Cialdini, 2003; Crum & Langer, 2007). In addition, how effective or competent a technology is at supporting educators or students can affect their trust and behavioral intentions according to TAM (perceived usefulness). The strong effect of framing different beneficiaries observed in prior work should motivate research in education to understand the impact of beneficiary framing of PLA on educators’ trust and their behavioral intentions.

Besides beneficiary framing, the increasing sophistication of AI models in education has raised questions about explainability, algorithm transparency, and trust (Cukurova et al., 2020; Khosravi et al., 2022). While shielding the end-user from the inner workings of the technology can provide a smooth user experience, it may also raise concerns especially among expert users. Behavioral decision science research highlights the critical importance of people’s expectations and algorithmic literacy for mitigating algorithm aversion (Burton et al., 2020). Educators using a PLA dashboard may want to understand how it concluded that some students are at-risk of failing a course, especially if the prediction does not align with their expectation. This may be achieved with explainable AI techniques and by improving algorithmic literacy (Long & Magerko, 2020). Most educators are not trained in AI and may experience algorithm aversion without a better understanding of how PLA works. There is at least one effort to create a professional development program for educators specifically about AI systems (Nazaretsky, Ariely et al., 2022). Even a simple explanation of an algorithm can increase algorithm transparency: learners in an online course showed more trust in a peer grading system that violated their expectations when an algorithm explanation was provided (Kizilcec, 2016), and a study of Bayesian Knowledge Tracing found that an explanation that provided algorithm transparency improved student attitudes towards the system (Williamson & Kizilcec, 2021). There is substantial room for expanding our understanding of how to design an effective intervention to increase algorithm transparency to promote educators’ trust and positive behavioral intentions. Moreover, PLA literacy interventions to help educators develop a theory of mind for how PLA works, its purpose, and how to leverage effectively to augment their own decision making should also be evaluated for their effectiveness.

## Focus on Understanding Educators

The successful implementation and effective use of AI systems in educational contexts is a sociotechnical challenge and therefore requires careful consideration of human factors (Buckingham Shum, Ferguson, & Martinez-Maldonado, 2019). Educators play an essential role in decisions about the adoption of AI systems, but they also have limited time and flexibility to engage in new activities. Technology designers need to understand the status quo of educators’ environment, workflow, schedule, and resources to identify entry points with a clear value proposition for educators’ return on investment for the time it takes to integrate a new tool. For example, many educators use tools like Google Docs and have experienced the gradual introduction of AI features based on large language models (e.g., rephrasing suggestions), which are easy to integrate into daily practice. Similarly, tools that fit seamlessly into ubiquitous learning management systems to help educators’ achieve effortful tasks, such as grading or summarizing student responses, are more likely to be adopted. A pragmatic view of technology adoption also highlights the role of cost and reliability of hardware and software; reliability is a particularly salient factor for any educator who has encountered issues during class time.

A better understanding of how educators perceive AI systems is necessary because educators are ultimately the final decision makers in this context. Even a perfectly reliable, accurate, and fair system is going to fail in practice if educators experience concerns about usability, errors, and algorithmic biases. Presently, we have a limited understanding of what different groups of educators think about different kinds of AIED systems, how well they understand key technologies underlying these systems, and whether they consider them helpful and trustworthy. Given the large diversity of systems, educators, and environments, this presents a large problem space for future research efforts to build a systematic understanding of the way that technology, educator, and context characteristics shape educators’ beliefs and attitudes. Future work that examines these issues causally can experimentally test interventions to shape educators’ beliefs and attitudes by varying the framing of AI systems, algorithm transparency and literacy, building on theoretical models of technology acceptance and academic resistance. The goal should not be to maximize educators’ trust in AIED systems, which may cause overreliance and an inadvertent loss in trust, but rather to create conditions under which educators can build up trust over time through experience. Over time, educators may begin to see AIED systems as if they were competent, reliable, and resourceful members of the teaching staff.

## References

[CR1] Adams, D. A., Nelson, R. R., & Todd, P. A. (1992). Perceived usefulness, ease of use, and usage of information technology: A replication. MIS quarterly, 227–247.

[CR2] Ames, M. G. (2019). *The charisma machine: The life, death, and legacy of one laptop per child*. MIT Press.

[CR3] Arnold, K. E., & Pistilli, M. D. (2012). Course signals at Purdue: Using learning analytics to increase student success. In *Proceedings of the 2nd international conference on learning analytics and knowledge* (pp. 267–270).

[CR4] Baker, R. S., Berning, A. W., Gowda, S. M., Zhang, S., & Hawn, A. (2020). Predicting K-12 dropout. *Journal of Education for Students Placed at Risk (JESPAR)*, *25*(1), 28–54.

[CR5] Bryant, J., Child, F., Dorn, E., & Hall, S. (2020). New global data reveal education technology’s impact on learning. McKinsey & Company blog. Available at https://www.mckinsey.com/industries/public-and-social-sector/our-insights/new-global-data-reveal-education-technologys-impact-on-learning

[CR6] Buckingham Shum, S., Ferguson, R., & Martinez-Maldonado, R. (2019). Human-centred learning analytics. *Journal of Learning Analytics*, *6*(2), 1–9.

[CR7] Burton, J. W., Stein, M. K., & Jensen, T. B. (2020). A systematic review of algorithm aversion in augmented decision making. *Journal of behavioral decision making*, *33*(2), 220–239.

[CR8] Christensen, C. M., Horn, M. B., & Johnson, C. W. (2008). How disruptive innovation will change the way we learn. *Education Week*, *27*(39), 25–36.

[CR9] Cialdini, R. B. (2003). Crafting normative messages to protect the environment. *Current directions in psychological science*, *12*(4), 105–109.

[CR10] Clary, E. G., & Snyder, M. (1999). The motivations to volunteer: Theoretical and practical considerations. *Current directions in psychological science*, *8*(5), 156–159.

[CR11] Crum, A. J., & Langer, E. J. (2007). Mind-set matters: Exercise and the placebo effect. *Psychological science*, *18*(2), 165–171. 10.1111/j.1467-9280.2007.01867.x 17425538

[CR12] Cuban, L. (2009). *Oversold and underused*. Harvard University Press.

[CR13] Cukurova, M., Luckin, R., & Kent, C. (2020). Impact of an artificial intelligence research frame on the perceived credibility of educational research evidence. *International Journal of Artificial Intelligence in Education*, *30*(2), 205–235.

[CR15] Davis, F. D. (1989). Perceived usefulness, perceived ease of use, and user acceptance of information technology. MIS quarterly, 319–340.

[CR14] Dietvorst, B. J., Simmons, J. P., & Massey, C. (2015). Algorithm aversion: People erroneously avoid algorithms after seeing them err. *Journal of experimental psychology: general*, *144*(1),114. 10.1037/xge0000033 25401381

[CR16] Fathema, N., Shannon, D., & Ross, M. (2015). Expanding the Technology Acceptance Model (TAM) to examine faculty use of Learning Management Systems (LMSs) in higher education institutions. *Journal of online learning & teaching*, *11*(2).

[CR17] Floridi, L., & Cowls, J. (2022). A unified framework of five principles for AI in society. Machine learning and the city: Applications in architecture and urban design, 535–545.

[CR18] Grant, A. M., & Hofmann, D. A. (2011). It’s not all about me: Motivating hand hygiene among health care professionals by focusing on patients. Psychological science, *22*(12), 1494–1499. 10.1177/0956797611419172 22075239

[CR20] Herodotou, C., Rienties, B., Boroowa, A., Zdrahal, Z., & Hlosta, M. (2019). A large-scale implementation of predictive learning analytics in higher education: The teachers’ role and perspective. *Educational technology research and development*, *67*(5), 1273–1306.

[CR19] Hlosta, M., Herodotou, C., Bayer, V., & Fernandez, M. (2021). Impact of predictive learning analytics on course awarding gap of disadvantaged students in stem. In *International Conference on Artificial Intelligence in Education* (pp. 190–195). Springer, Cham.

[CR22] Khosravi, H., Shum, S. B., Chen, G., Conati, C., Tsai, Y. S., Kay, J., & Gašević, D. (2022). Explainable Artificial Intelligence in education. *Computers and education: artificial intelligence*, 3, 100074.

[CR23] Kizilcec, R. F. (2016). How much information? Effects of transparency on trust in analgorithmic interface. In *Proceedings of the 2016 CHI conference on human factors in computing systems* (pp. 2390–2395).

[CR24] Kurzweil, M., & Wu, D. D. (2015). Building a pathway to student success at Georgia State University. GHPC Articles, 142.

[CR25] Kuzilek, J., Hlosta, M., Herrmannova, D., Zdrahal, Z., Vaclavek, J., & Wolff, A. (2015). OU Analyse: Analysing at-risk students at the Open University. *Learning analytics review*, 1–16.

[CR26] Lee, Y., Kozar, K. A., & Larsen, K. R. (2003). The technology acceptance model: Past, present, and future. *Communications of the association for information systems*, *12*(1),50.

[CR27] Long, D., & Magerko, B. (2020). What is AI literacy? Competencies and design considerations. In *Proceedings of the 2020 CHI conference on human factors in computing systems* (pp. 1–16).

[CR28] Nazaretsky, T., Ariely, M., Cukurova, M., & Alexandron, G. (2022a). Teachers’ trust in AI-powered educational technology and a professional development program to improve it. British journal of educational technology.

[CR29] Nazaretsky, T., Cukurova, M., & Alexandron, G. (2022b). An Instrument for Measuring Teachers’ Trust in AI-Based Educational Technology. In LAK22: 12th international learning analytics and knowledge conference (pp. 56–66).

[CR30] Park, S. Y. (2009). An analysis of the technology acceptance model in understanding university students’ behavioral intention to use e-learning. *Journal of educational technology & society*, *12*(3), 150–162.

[CR31] Piderit, S. K. (2000). Rethinking resistance and recognizing ambivalence: A multidimensional view of attitudes toward an organizational change. *The Academy of Management Review*, *25*(4), 783–794.

[CR32] Reich, J. (2020). Failure to disrupt: Why technology alone can’t transform education. Harvard University Press.

[CR33] Reich, J., & Mehta, J. (2020). Imagining September: Principles and design elements for ambitious schools during COVID-19. 10.35542/osf.io/gqa2w

[CR34] Rienties, B. (2014). Understanding academics’ resistance towards (online) student evaluation. *Assessment & Evaluation In Higher Education*, *39*(8), 987–1001.

[CR35] Rothman, A. J., & Salovey, P. (1997). Shaping perceptions to motivate healthy behavior: The role of message framing. *Psychological bulletin*, *121*(1), 3. 10.1037/0033-2909.121.1.3 9000890

[CR36] Teo, T. (2009). Modelling technology acceptance in education: A study of pre-service teachers. *Computers & education*, *52*(2), 302–312.

[CR37] Venkatesh, V., & Bala, H. (2008). Technology acceptance model 3 and a research agenda on interventions. *Decision sciences*, *39*(2), 273–315.

[CR38] Venkatesh, V., & Davis, F. D. (2000). A theoretical extension of the technology acceptance model: Four longitudinal field studies. *Management science*, *46*(2), 186–204.

[CR39] Williamson, K., & Kizilcec, R. F. (2021). Effects of Algorithmic Transparency in Bayesian Knowledge Tracing on Trust and Perceived Accuracy. *Intl. conference on educational data mining*

[CR40] Yan, L., Sha, L., Zhao, L., Li, Y., Martinez-Maldonado, R., Chen, G., Li, X., Jin, Y., & Gašević, D. (2023). Practical and ethical challenges of large language models in education: A systematic literature review. *arXiv preprint arXiv:2303.13379*

[CR41] Yi, M. Y., & Hwang, Y. (2003). Predicting the use of web-based information systems: Self-efficacy, enjoyment, learning goal orientation, and the technology acceptance model. International journal of human-computer studies.

[CR42] Zawacki-Richter, O., Marín, V. I., Bond, M., & Gouverneur, F. (2019). Systematic review of research on Artificial Intelligence applications in higher education–where are the educators?. *International journal of educational technology in higher education*, *16*(1), 1–27.

